# Prevalence of Hepatitis C Virus Antibody in Patients With Sexually Transmitted Diseases Attending a Harrisburg, PA, STD Clinic

**DOI:** 10.1155/S1064744994000232

**Published:** 1994

**Authors:** Robert L. Sautter, Sharon Jones, Daniel I. Weber, William D. LeBar, Daniel F. Heitjan, Mary Magdalene C. Kopreski, Frederick D. Curcio

**Affiliations:** 1 Department of Microbiology and Pathology Harrisburg Hospital, South Front Street Harrisburg, Hershey Medical Center The Pennsylvania State University Hershey PA 17101 USA; 2 Department of Obstetrics and Gynecology Harrisburg Hospital Harrisburg, Hershey Medical Center The Pennsylvania State University Hershey PA USA; 3 Planned Parenthood of the Capital Region Harrisburg, Hershey Medical Center The Pennsylvania State University Hershey PA USA; 4 Department of Obstetrics and Gynecology Hershey Medical Center The Pennsylvania State University Hershey PA USA; 5 Center for Biostatistics and Epidemiology Hershey Medical Center The Pennsylvania State University Hershey PA USA; 6 Department of Pathology Hershey Medical Center The Pennsylvania State University Hershey PA USA; 7 Department of Microbiology and Pathology Citation Clinical Laboratory-Providence Hospital Southfield MI USA

## Abstract

*Objective:* The prevalence of hepatitis B and hepatitis C in a sexually transmitted disease (STD) clinic population was studied, along with the prevalence of various STD agents, in an attempt to identify possible STD markers for the hepatitis C virus and help delineate the role of hepatitis C as an STD. The hepatitis C antibody rates found in the STD clinic were also compared with those found among patients attending a local OB/GYN clinic and those enrolled in a blood donor program, all from the same geographical area.

*Methods:* A total of 150 women attending an STD clinc were examined for each of the following agents: *Chlamyadia trachomatis*, *Neisseria gonorrhoeae*, syphilis, hepatitis B surface antigen, hepatitis B core antibody, hepatitis B surface antibody, and hepatitis C virus antibody. Additionally, several patients who signed informed consent to be evaluated for human immunodeficiency virus (HIV) antibody were tested by an enzyme immunoassay (EIA) screen method. The prevalence of each agent was then compared with the other agents.

*Results:* The overall prevalence rates detected were as follows: hepatitis B 16%, hepatitis C 4%, chlamydia 18.7%, gonorrhea 7.4%, syphilis 0.7%, and HIV 0%. Hepatitis C antibody was detected in 4% of patients in the STD clinic, 0.76% of volunteer blood donors from central  Pennsylvania, and 0% of patiants studied from the Harrisburg Hospital (Harrisburg, PA) prentatal population.

*Conclusions:* This screening study reveals an association between attending a Harrisburg, PA, area STD clinic and having an increased prevalence of hepatitis C antibody, but larger matched control studies will be needed to help clarify sexual transmission as a mode of transmission for the hepatitis C virus.


Infectious Diseases in Obstetrics and Gynecology 1:269–274 (1994)
© 1994 Wiley-Liss, Inc.

### Prevalence of Hepatitis C Virus Antibody in Patients With Sexually Transmitted Diseases Attending a Harrisburg, PA, STD Clinic

**Robert L. Sautter, Sharon Jones, Daniel I. Weber, William D. LeBar, Daniel F. Heitjan, Mary Magdalene C. Kopreski, and Frederick D. Curcio**

*Departments of Microbiology and Pathology (R.L.S.) and Obstetrics and Gynecology (D.I.W., F.D.C.), Harrisburg Hospital, and Planned Parenthood of the Capital Region (S.J., D.I.W.), Harrisburg, and Department of Obstetrics and Gynecology (D.I.W., M.M.C.K., F.D.C.), Center for Biostatistics and Epidemiology (D.F.H.), and Department of Pathology (R.L.S.), Hershey Medical Center, The Pennsylvania State University, Hershey, PA; and Department of Microbiology and Pathology (W.D.L.), Citation Clinical Laboratory-Providence Hospital, Southfield, MI*

### Abstract

***Objective:*** The prevalence of hepatitis B and hepatitis C in a sexually transmitted disease (STD) clinic population was studied, along with the prevalence of various STD agents, in an attempt to identify possible STD markers for the hepatitis C virus and help delineate the role of hepatitis C as an STD. The hepatitis C antibody rates found in the STD clinic were also compared with those found among patients attending a local OB/GYN clinic and those enrolled in a blood donor program, all from the same geographical area.

***Methods:*** A total of 150 women attending an STD clinic were examined for each of the following agents: ***Chlamydia trachomatis, Neisseria gonorrhoeae,*** syphilis, hepatitis B surface antigen, hepatitis B core antibody, hepatitis B surface antibody, and hepatitis C virus antibody. Additionally, several patients who signed informed consent to be evaluated for human immunodeficiency virus (HIV) antibody were tested by an enzyme immunoassay (EIA) screen method. The prevalence of each agent was then compared with the other agents.

***Results:*** The overall prevalence rates detected were as follows: hepatitis B 16%, hepatitis C 4%, chlamydia 18.7%, gonorrhea 7.4%, syphilis 0.7%, and HIV 0%. Hepatitis C antibody was detected in 4% of patients in the STD clinic, 0.76% of volunteer blood donors from central Pennsylvania, and 0% of patients studied from the Harrisburg Hospital (Harrisburg, PA) prenatal population.

***Conclusions:*** This screening study reveals an association between attending a Harrisburg, PA, area STD clinic and having an increased prevalence of hepatitis C antibody, but larger matched control studies will be needed to help clarify sexual transmission as a mode of transmission for the hepatitis C virus. © 1994 Wiley-Liss, Inc.

Key words
Incidence, non-A, non-B hepatitis, STD

Post-transfusion hepatitis is reported in 10–20% of patients receiving 3 or more units of blood. Many patients who contract hepatitis have no detectable antibody against type A or B hepatitis viruses and are classified as having non-A, non-B hepatitis. Ninety percent of post-transfusion hepa-

Address correspondence/reprint requests to Dr. Robert L. Sautter, Departments of Microbiology and Pathology, Harrisburg Hospital, South Front Street, Harrisburg, PA 17101.

Received March 7, 1994

Accepted May 12, 1994

Clinical Study titis has now been attributed to non-A, non-B hepatitis worldwide.[1,2] However, this type of transmission was recently estimated to account for as low as only 10–15% of patients with non-A, non-B hepatitis.[3] Recently, the isolation and cloning of a piece of DNA from non-A, non-B hepatitis virus and development of an assay for the antibody to hepatitis C virus (HCV) made possible the detection of many patients with a non-A, non-B hepatitis and the examination of transmission routes.[4,5] Recently, a 2nd-generation test for the detection of antibody vs. HCV was licensed.[6] This 2nd-generation test offers the advantage of increased sensitivity and specificity for the determination of HCV antibody.[5,6] Results have suggested that HCV is the major cause of transfusion-related non-A, non-B hepatitis,[7] especially in those cases that develop chronicity.[8] Additionally, HCV appears to be the major cause of a number of community-acquired non-A, non-B hepatitis for which no history of percutaneous exposure has been identified.[1,2,9]

Studies investigating the possible sources of infection for non-A, non-B hepatitis or HCV without a history of percutaneous exposure have been contradictory to date. Several small case reports have been published recognizing possible transmission due to perinatal and conjugal relationships that follow patterns similar to transmission of hepatitis B, human immunodeficiency virus (HIV), and human T-lymphotropic virus type I (HTLV 1).[10,11] In addition, other papers including studies relating HCV to patients with sexually transmitted diseases (STDs)[6,12] and to heterosexual activity with more than 1 partner have been published.[13] Contrary to these findings, other investigations have suggested only rare sexual transmission of HCV among homosexuals[3] and among sexual contacts of high-risk intravenous (IV)-drug abusers.[2]

To further delineate the possible method of spread for HCV, we studied the prevalence of hepatitis B infection and hepatitis C infection in an STD clinic population and correlated other known STDs as possible markers for patients at high risk for hepatitis B and hepatitis C.

### SUBJECTS AND METHODS

### Subjects

Women attending a Harrisburg area STD clinic were included in the study if they signed informed consent and completed a questionnaire assessing their risk factors (Table 1). Black, Caucasian, and Hispanic individuals were included in the study. The prevalence of hepatitis C in the central Pennsylvania blood donor population and the Harrisburg Hospital prenatal population was also evaluated.

**TABLE 1. Demographic and clinical information collected on STD patients**

| | | |
|---|---|---|
| Today's date: Age: | | |
| 1. Race (circle one): | | |
| Black White Hispanic Asian Other | | |
| 2. Did you have a blood transfusion (received blood) between 1979 and May 1985? | Yes | No |
| 3. Has there been any one year since 1980 during which you had more than 5 partners? | Yes | No |
| 4. In the past 9 years, have you had sex with a person who was | | |
| A. an IV drug abuser | Yes | No |
| B. a hemophiliac | Yes | No |
| C. a bisexual | Yes | No |
| D. a prostitute | Yes | No |
| 5. Have you ever had sex with anyone who (to your knowledge) had AIDS or was infected with the AIDS virus? | Yes | No |
| 6. Have you ever had any of the following STDs? | | |
| Gonorrhea | Yes | No |
| Herpes | Yes | No |
| Chlamydia | Yes | No |
| Syphilis | Yes | No |
| Genital warts | Yes | No |
| Pelvic inflammatory disease | Yes | No |
| 7. In the past 9 years, have you used IV drugs? | Yes | No |
| 8. Have you ever had sex in exchange for money or drugs? | Yes | No |

### Laboratory Methods

#### *Chlamydia trachomatis*

Two direct antigen tests (enzyme immunoassay [EIA] methods, Chlamydiazyme, Abbott Laboratories, Abbott Park, IL, and a research membrane filtration technique, Seradyn, Inc., Indianapolis, IN) and culture were performed on the specimens collected from the patients for the diagnosis of infection with *C. trachomatis*. In addition, the culture transport fluid was analyzed by direct immunofluorescence (DFA) for the presence of *C. trachomatis* antigen as previously described.[14] A specimen was considered positive for *C. trachomatis* if the culture was positive or if 2 of the 3 direct antigen tests were positive. All chlamydial procedures were performed according to the manufacturers' specifications.

**TABLE 2. Percentage of patients exhibiting multiple risk factors**

| Total number of risk factors | % |
|---|---|
| 0 | 35 |
| 1 | 37 |
| 2 | 17 |
| 3 | 6 |
| 4 | 4 |
| 5 | 0 |
| 6 | 1 |
| Total | 100 |

### Hepatitis B Testing

Auszyme, hepatitis BsAg (HBsAg), Corzyme, anti-hepatitis B core antigen (anti-HBc), and Ausab, anti-hepatitis Bs antigen (anti-HBs; Abbott Laboratories) were performed according to the manufacturer's specifications. Only those specimens that were repeatedly reactive were classified positive. For statistical analysis, those patients who exhibited 1 or all of the above markers without history of vaccination were considered to have evidence of hepatitis B infection at some time in the past.

### Hepatitis C Testing

The presence of serum HCV (anti-HCV; Abbott Laboratories) antibody was measured according to the manufacturer's specifications. Both 1st- and 2nd-generation tests for the detection of antibody vs. HCV were used. Each reactive result was confirmed in duplicate and sent for confirmatory testing. The confirmatory test performed was the Chiron HCV recombinant immunoblot assay (RIBA; Chiron Corporation, Berkeley, CA).

### Bacterial Culture and Syphilis Serology

Gonococcal cultures were performed on Martin Lewis agar medium in a 5% carbon dioxide atmosphere. Standard bacteriologic techniques were used to identify the isolates.[15] Syphilis serology utilized a standard Rapid Plasma Reagin (RPR) assay.[16]

### Statistical Analysis

The goal of our analyses was to determine whether there was a significant association between the STDs, i.e., whether presence of 1 STD increased the chance of having another. Thus, every pair of STDs was tested for association using the Fisher-Irwin exact test (Table 2).[17] To determine whether the observed associations would hold up after controlling for STD risk status, we classified the women into high-risk and low-risk strata, then carried out a stratified analysis using the Cochran-Mantel-Haenszel (CMH) test.[18] We determined risk status by questionnaire and chart review (Table 3). Using these criteria, we called subjects "low risk" if they had no known behavioral or medical risk factors and classified those subjects with any risk factors as "high risk." Most computations were executed in the S-Plus language on a Sun SPARC-station 1 workstation.[19] Exact tests were computed with StatXact, version 2.0 (Cytel, Cambridge, MA).

**TABLE 3. Total number of patients categorized by questionnaire and chart review for demographic data and risk characteristics**

| | |
|---|---|
| Race | % |
| Hispanic | 4 |
| Caucasian | 49 |
| Black | 46 |
| Asian | 0 |
| Other | 1 |
| Total | 100 |
| Age (years) | |
| 13–20 | 45 |
| 21–30 | 37 |
| 31–40 | 14 |
| 41–50 | 3 |
| 51–54 | 1 |
| Total | 100 |
| High-risk sexual practices | |
| Multiple sex partners | 17 |
| With IV-drug user | 10 |
| With hemophiliac | 0 |
| With bisexual | 4 |
| With prostitute | 1 |
| With someone having AIDS | 1 |
| For money or drugs | 2 |
| Previous medical conditions | |
| Blood transfusion | 2 |
| Gonorrhea | 19 |
| Syphilis | 3 |
| Herpes | 3 |
| Genital warts | 19 |
| Chlamydia | 23 |
| Pelvic inflammatory disease | 6 |

### RESULTS

Demographic information on patients seen at the Harrisburg area STD clinic is presented in Table 3. Subjects ranged in age from 13 to 54 years. The most frequent risk factors are presented as percentages in Table 3 and by prevalence in Table 2. The most prevalent risk factor was multiple sexual partners—26 (17%), followed by sex with an IV-drug

TABLE 4. Prevalence rates of 6 STDs with *P* values for tests of association[a]

| Disease (prevalence) | *P* value from test of association | | | | |
|---|---|---|---|---|---|
| | Hepatitis C | Chlamydia | Gonorrhea | Syphilis | HIV |
| Hepatitis B (16.0%) | 0.052 | 0.701 | 0.557 | 0.327 | ND |
| Hepatitis C (4.0%) | | 0.717 | 0.063 | 0.082 | ND |
| Chlamydia (18.7%) | | | 0.034 | 0.189 | ND |
| Gonorrhea (7.4%) | | | | 0.151 | ND |
| Syphilis (0.7%) | | | | | ND |
| HIV (0%) | | | | | |

[a]*P* values are from 1-sided Fisher exact tests of the hypothesis of no association between the diseases against the alternative of positive association. ND indicates insufficient data to test the hypothesis. Sample sizes are 149 for gonorrhea, 148 for syphilis, 27 for HIV, and 150 for hepatitis B and C and chlamydia.

abuser—15 (10%). Thirty-five patients admitted to having had a chlamydial infection at some time in the past. There were 53 "low-risk" and 97 "high-risk" subjects as defined in the previous section. Prevalence rates of 6 STDs are presented in Table 4. When the 1-sided Fisher-Irwin test was performed, associations between chlamydia and gonorrhea ($P = 0.034$) and between hepatitis B and hepatitis C ($P = 0.052$) were found. After stratification using the CMH test, the chlamydia/gonorrhea test ($P = 0.052$) and the hepatitis B/hepatitis C test ($P = 0.086$) approach significant positive associations. These results support conclusions reached using the Fisher-Irwin test mentioned earlier. The only statistically significant association following stratification was found between hepatitis C and syphilis ($P = 0.044$). However, the association between these 2 diseases was negative.

Seven patients were found to be repeatedly reactive by the HCV EIA procedure. Six of the 7 (85.7%) reactive EIA specimens were found to be positive for antibody to HCV by the 2nd-generation HCV (EIA) procedure and by the RIBA. Of these 6 patients, 3 were also positive for hepatitis B core antibody. Of the subjects who were confirmed positive for HCV antibody, only 1 (16%) had no risk factor as defined earlier. Three or 50% of the HCV-positive subjects had multiple risk factors with the most common risk factors being previous blood transfusion (50%), IV-drug abuse (33%), and multiple sexual partners (33%). No statistically significant associations were found for HCV-positive subjects and their risk factors. Other STDs were detected in those patients positive for HCV; however, no statistically significant association was determined. The overall prevalence of hepatitis C in 3 populations (STD, prenatal, and blood donor) is presented in Table 5. They are significantly different by the exact test on the $3 \times 2$ table ($P = 0.0005$). Pairwise differences are significant for STD clinic vs. blood donor ($P = 0.0012$) and STD clinic vs. prenatal ($P = 0.04$). The blood donor and the prenatal groups are not significantly different.

TABLE 5. Comparison of hepatitis C antibody in 3 Harrisburg patient populations[a]

| Population studied | Prevalence |
|---|---|
| STD clinic | 6/150 (4%) |
| Blood donor | 59/7,744 (0.76%) |
| Prenatal | 0/100 (0%) |

[a]All positive HCV antibody tests were confirmed by RIBA.

### DISCUSSION

HCV has been shown to be the causative agent of the majority of cases of post-transfusion hepatitis,[1,20,21] especially high-risk transfusion patients such as hemophiliacs,[22–24] chronic renal patients, and those patients with recent cardiac surgery.[24] In addition, the agent has been found in U.S. veterans,[25] and implicated in maternal transmission,[11,26] sexual transmission,[12,13,27] and IV-drug abuse.[28,29] The purpose of the present study was to explore the relationships between STDs and current or prior HCV infection and thus identify known STDs as possible markers for HCV. To discriminate the prevalence of HCV in the high-risk groups from that in the normal population, we studied the prevalence of HCV in 2 low-risk patient populations in the Harrisburg area.

Positive associations between STDs were found in the 150 STD patients studied. As expected, patients positive for *C. trachomatis* were likely to be infected with *N. gonorrhoeae*. We also found hepatitis B virus (HBV) and HCV to be associated with one another. The presence of hepatitis B markers (anti-HBc, anti-HBe, HBeAg) has been related to the presence of HCV in blood donors and chronic HCV carriers.[30,31] However, significant debate continues on the reliability of surrogate markers in blood donor populations for predicting the presence of HCV.[32–34]

HBV and HCV seem to be transmitted concomitantly in the United States and most of Europe, while Japan and selected countries in Europe show little or no association between transmission of HBV and HCV. Differences in these associations could possibly be due to some unknown risk factors that are found in certain geographical locales and not in others. It has been suggested that several classes of HCV exist, with varying subtypes more prevalent in different countries. Geographical and/or genetic differences have yet to be explored as a method for interpreting transmission routes and prevalence rates. Studies have also suggested that heterosexual promiscuity and/or homosexual promiscuity with evidence of numerous prior STDs constitute significant risk factors for the transmission of both HBV and HCV.[12,13,27] Considerable debate over the role sexual practices have on the transmission of HCV can be found in the current literature.[3,9]

In the present study, antibody to HCV was detected in 4% of patients attending a Harrisburg area STD clinic, in 0.76% of volunteer blood donors from central Pennsylvania, and in none of the patients studied from Harrisburg Hospital's prenatal population. Hess et al.[9] found similar results with 4.7% and 0.51% positive anti-HCV results from STD and blood donor patients, respectively. Additional studies in the current literature have shown that the positive rates for HCV in blood donors range between 0.5 and 1.5%[1,21,34,35] Positive rates for sexual transmission of non-A, non-B hepatitis in heterosexual and homosexual populations have ranged between 4.7 and 50%.[9,12,13,27,35] Additional risk factors have included race, nationality, sex of the patient, multiple sexual partners, IV-drug abuse, previous or concurrent positive tests for HBV and HIV, and evidence of multiple STDs. Our data differ slightly from a recently published review by Lynch-Salamon and Combs.[35] The incidence reported in their review of the literature agrees with our data for blood donors and for those patients attending an STD clinic. However, the incidence of HCV positivity by risk groups in our study is lower than that previously reported.[35] This is undoubtedly due to the small number of HCV-positive patients found in the Harrisburg area STD clinic. The present study shows that attending a Harrisburg area STD clinic is associated with an increased prevalence of HCV compared with 2 other low-risk populations in the same geographical area. However, we were unable to identify any specific disease among the known STDs that correlated statistically with the presence of HCV for use as a marker for HCV infection. Additional larger studies involving matched controls would be helpful in order to help clarify the mode of transmission of HCV.

### ACKNOWLEDGMENTS

We thank the Harrisburg Hospital laboratory staff in the Department of Microbiology and Blood Bank as well as the staff of Planned Parenthood of the Capital Region for their technical assistance. This work was supported by a George Lafferty Foundation grant. Supplies for hepatitis screening were provided by Abbott Laboratories (Abbott Park, IL).

Additionally, we dedicate this paper to the late Dr. Frederick D. Curcio III, whose intellect, guidance, friendship, and compassion will be remembered by all those individuals who have benefited from knowing this extremely caring individual.

### REFERENCES

1. Stevens CE, Taylor PE, Pindyck J, et al.: Epidemiology of hepatitis C virus, a preliminary study in volunteer blood donors. JAMA 263:49–53, 1990.
2. Esteban JI, Viladomiu L, Gonzalez A, et al.: Hepatitis C virus antibodies among risk groups in Spain. Lancet 2:294–296, 1989.
3. Melbye M, Biggar RJ, Wantzin P, Krogsgard K, Ebbesen P, Becker NG: Sexual transmission of hepatitis C virus: Cohort study (1981–9) among European homosexual men. Br Med J 301:210–212, 1990.
4. Choo QL, Kuo G, Weiner AJ, Overby LR: Isolation of a cDNA clone derived from a blood-borne non-A, non-B viral hepatitis genome. Science 244:359–361, 1989.
5. Kuo G, Choo Q-L, Alter HJ, Citnick GL: An assay for circulating antibody to a major etiologic virus of human NANB hepatitis. Science 244:362–364, 1989.
6. Aach RD, Stevens CE, Hollinger FB, Moseley JW: Hepatitis C virus infection in post-transfusion. An analysis with first- and second-generation assays. N Engl J Med 325:1325–1329, 1991.

7. Marcellin P, Martinot-Peignoux M, Boyer N, et al.: Second-generation (RIBA) test for hepatitis C virus (letter). Lancet 337(8740):551–552, 1991.
8. Mosely JW, Aach RD, Hollinger FB, et al.: Non-A, non-B hepatitis and antibody to hepatitis C virus. JAMA 263:77–78, 1990.
9. Hess G, Massing A, Rossol S, et al.: Hepatitis C virus and sexual transmission. Lancet 2:987, 1989.
10. Kamitsukasa H, Harada H, Yakura M, et al.: Intrafamilial transmission of hepatitis C virus (letter). Lancet 2:987, 1989.
11. Kuroki T, Nishiguchi S, Fukuda K, et al.: Mother-to-child transmission of hepatitis C virus. J Infect Dis 164: 427–428, 1991.
12. Tedder RS, Gilson RJC, Briggs M, et al.: Hepatitis C virus: Evidence for sexual transmission. Br Med J 302: 1299–1302, 1991.
13. Alter MJ, Coleman PJ, Alexander WJ, et al.: Importance of heterosexual activity in the transmission of hepatitis B and non-A, non-B hepatitis. JAMA 262:1201–1205, 1989.
14. LeBar WD, Schubiner H, Jemal C, et al.: Comparison of the Kallestad Pathfinder EIA, cytocentrofuged direct fluorescent antibody, and cell culture for the detection of *Chlamydia trachomatis*. Diagn Microbiol Infect Dis 14: 17–20, 1991.
15. Morello JA, Janda W, Doern GV: *Neisseria* and *Branhamella*. In Balows A (ed): Manual of Clinical Microbiology. 5th ed. Washington, DC: American Society for Microbiology, pp 258–276, 1991.
16. Larsen SA, Bradford LL: Serodiagnosis of syphilis. In Rose NR (ed): Manual of Clinical Laboratory Immunology. 3rd ed. Washington, DC: American Society for Microbiology, pp 425–434, 1986.
17. Placket RL: The Analysis of Categorical Data. 2nd ed. New York: Macmillan, 1981.
18. Snedecor GW, Cochran WG: Statistical Methods. 9th ed. Ames: Iowa State University Press, 1989.
19. Statistical Sciences, Inc.: S Plus Version 2.3. Seattle: Statistical Sciences, Inc., 1990.
20. Lee SH, Hwang SJ, Lu RH, Lai KH, Tsai YT, Lo KJ: Antibodies to hepatitis C virus in prospectively followed patients with posttransfusion hepatitis. J Infect Dis 163: 1354–1357, 1991.
21. Goldsmith MF: Blood bank officials hope donor altruism will pass new (anti-HCV) test. JAMA 262:1749–1750, 1990.
22. Widell A, Hansson BG, Bertorp E, et al.: Antibody to a hepatitis C virus related protein among patients at high risk for hepatitis B. Scand J Infect Dis 23:19–24, 1991.
23. Schulmn S, Grillner L: Antibodies against hepatitis C in a population of Swedish haemophiliacs and heterosexual partners. Scand J Infect Dis 22(4):393–397, 1990.
24. Brind AM, Codd AA, Cohen BJ, et al.: Low prevalence of antibody to hepatitis C virus in northeast England. J Med Virol 32(4):243–248, 1990.
25. Kendrick V, Dunn B, Fink L, et al.: The presence of hepatitis C virus in veterans with and without abnormal liver function. Am J Clin Pathol (Abstr Fall Meet No 79) 96:419, 1991.
26. Giovannini M, Tagger A, Ribero ML, et al.: Maternal-infant transmission of hepatitis C virus and HIV infections: A possible interaction (letter). Lancet 335:1166, 1990.
27. Perillo RP: Potential importance of the sexual transmission of non-A, non-B hepatitis. Hepatology 13:805–808, 1991.
28. van den Hoek JAR, van Haastrecht HJA, Goudsmit J, deWolf F, Coutinho RA: Prevalence, incidence, and risk factors of hepatitis C virus infection among drug users in Amsterdam. J Infect Dis 162:823–826, 1990.
29. Girardi E, Zaccarelli M, Tossini G, Puro V, Narciso P, Visco G: Hepatitis C virus infection to intravenous drug users: Prevalence and risk factors. Scand J Infect Dis 22(5):751–752, 1990.
30. Fattovich G, Tagger A, Brollo L, et al.: Hepatitis C virus infection in chronic hepatitis B carriers. J Infect Dis 163:400–402, 1991.
31. Koziol DE, Holland PV, Alling DW, et al.: Antibody to hepatitis B core antigen as a paradoxical marker for non-A, non-B hepatitis agents in donated blood. Ann Intern Med 104:488–495, 1986.
32. Ohto H, Nomura H, Ohmura K, Ishijima A, Okazaki S: Low overlap between anti-HCV and anti-HBc in Japanese. Transfusion 31:88–89, 1991.
33. Hetland G, Skaug K, Larsen J, Maland A, Stromme JH, Storvold G: Prevalence of anti-HCV in Norwegian blood donors with anti-HBc or increased ALT levels. Transfusion 30:776–779, 1990.
34. Richards P, Holland P, Kuramoto K, Dourville C, Randell R: Prevalence of antibody to hepatitis C virus in a blood donor population. Transfusion 31:109–113, 1991.
35. Lynch-Salamon DI, Combs CA: Hepatitis C in obstetrics and gynecology. Obstet Gynecol 79:621–629, 1992.

## References

[PDF_00470] Aach R. D., Stevens C. E., Hollinger F. B., Mosley J. W., Peterson D. A., Taylor P. E., Johnson R. G., Barbosa L. H., Nemo G. J. (1991). Hepatitis C virus infection in post-transfusion hepatitis. An analysis with first- and second-generation assays.. N Engl J Med.

[PDF_00493] Alter M. J., Coleman P. J., Alexander W. J., Kramer E., Miller J. K., Mandel E., Hadler S. C., Margolis H. S. (1989). Importance of heterosexual activity in the transmission of hepatitis B and non-A, non-B hepatitis.. JAMA.

[PDF_00464] Choo Q. L., Kuo G., Weiner A. J., Overby L. R., Bradley D. W., Houghton M. (1989). Isolation of a cDNA clone derived from a blood-borne non-A, non-B viral hepatitis genome.. Science.

[PDF_00457] Esteban J. I., Esteban R., Viladomiu L., López-Talavera J. C., González A., Hernández J. M., Roget M., Vargas V., Genescà J., Buti M. (1989). Hepatitis C virus antibodies among risk groups in Spain.. Lancet.

[PDF_00551] Fattovich G., Tagger A., Brollo L., Giustina G., Pontisso P., Realdi G., Alberti A., Ruol A. (1991). Hepatitis C virus infection in chronic hepatitis B virus carriers.. J Infect Dis.

[PDF_00536] Giovannini M., Tagger A., Ribero M. L., Zuccotti G., Pogliani L., Grossi A., Ferroni P., Fiocchi A. (1990). Maternal-infant transmission of hepatitis C virus and HIV infections: a possible interaction.. Lancet.

[PDF_00547] Girardi E., Zaccarelli M., Tossini G., Puro V., Narciso P., Visco G. (1990). Hepatitis C virus infection in intravenous drug users: prevalence and risk factors.. Scand J Infect Dis.

[PDF_00520] Goldsmith M. F. (1990). Blood bank officials hope donor altruism will pass new (anti-HCV) test.. JAMA.

[PDF_00482] Hess G., Massing A., Rossol S., Schütt H., Clemens R., Meyer zum Büschenfelde K. H. (1989). Hepatitis C virus and sexual transmission.. Lancet.

[PDF_00561] Hetland G., Skaug K., Larsen J., Maland A., Strømme J. H., Størvold G. (1990). Prevalence of anti-HCV in Norwegian blood donors with anti-HBc or increased ALT levels.. Transfusion.

[PDF_00484] Kamitsukasa H., Harada H., Yakura M., Fukuda A., Ohbayashi A., Saito I., Miyamura T., Choo Q. L., Houghton M., Kuo G. (1989). Intrafamilial transmission of hepatitis C virus.. Lancet.

[PDF_00554] Koziol D. E., Holland P. V., Alling D. W., Melpolder J. C., Solomon R. E., Purcell R. H., Hudson L. M., Shoup F. J., Krakauer H., Alter H. J. (1986). Antibody to hepatitis B core antigen as a paradoxical marker for non-A, non-B hepatitis agents in donated blood.. Ann Intern Med.

[PDF_00467] Kuo G., Choo Q. L., Alter H. J., Gitnick G. L., Redeker A. G., Purcell R. H., Miyamura T., Dienstag J. L., Alter M. J., Stevens C. E. (1989). An assay for circulating antibodies to a major etiologic virus of human non-A, non-B hepatitis.. Science.

[PDF_00487] Kuroki T., Nishiguchi S., Fukuda K., Shiomi S., Monna T., Murata R., Isshiki G., Hayashi N., Shikata T., Kobayashi K. (1991). Mother-to-child transmission of hepatitis C virus.. J Infect Dis.

[PDF_00497] LeBar W., Schubiner H., Jemal C., Herschman B., Criswell K., Curtin N., Sherbeck M. (1991). Comparison of the Kallested Pathfinder EIA, cytocentrifuged direct fluorescent antibody, and cell culture for the detection of Chlamydia trachomatis.. Diagn Microbiol Infect Dis.

[PDF_00516] Lee S. D., Hwang S. J., Lu R. H., Lai K. H., Tsai Y. T., Lo K. J. (1991). Antibodies to hepatitis C virus in prospectively followed patients with posttransfusion hepatitis.. J Infect Dis.

[PDF_00568] Lynch-Salamon D. I., Combs C. A. (1992). Hepatitis C in obstetrics and gynecology.. Obstet Gynecol.

[PDF_00476] Marcellin P., Martinot-Peignoux M., Boyer N., Pouteau M., Aumont P., Erlinger S., Benhamou J. P. (1991). Second generation (RIBA) test in diagnosis of chronic hepatitis C.. Lancet.

[PDF_00460] Melbye M., Biggar R. J., Wantzin P., Krogsgaard K., Ebbesen P., Becker N. G. (1990). Sexual transmission of hepatitis C virus: cohort study (1981-9) among European homosexual men.. BMJ.

[PDF_00479] Mosley J. W., Aach R. D., Hollinger F. B., Stevens C. E., Barbosa L. H., Nemo G. J., Holland P. V., Bancroft W. H., Zimmerman H. J., Kuo G. (1990). Non-A, non-B hepatitis and antibody to hepatitis C virus.. JAMA.

[PDF_00558] Ohto H., Nomura H., Ohmura K., Ishijima A., Okazaki S. (1991). Low overlap between anti-HCV and anti-HBc in Japanese.. Transfusion.

[PDF_00540] Perillo R. P. (1991). Potential importance of the sexual transmission of non-A, non-B hepatitis.. Hepatology.

[PDF_00565] Richards C., Holland P., Kuramoto K., Douville C., Randell R. (1991). Prevalence of antibody to hepatitis C virus in a blood donor population.. Transfusion.

[PDF_00526] Schulman S., Grillner L. (1990). Antibodies against hepatitis C in a population of Swedish haemophiliacs and heterosexual partners.. Scand J Infect Dis.

[PDF_00454] Stevens C. E., Taylor P. E., Pindyck J., Choo Q. L., Bradley D. W., Kuo G., Houghton M. (1990). Epidemiology of hepatitis C virus. A preliminary study in volunteer blood donors.. JAMA.

[PDF_00490] Tedder R. S., Gilson R. J., Briggs M., Loveday C., Cameron C. H., Garson J. A., Kelly G. E., Weller I. V. (1991). Hepatitis C virus: evidence for sexual transmission.. BMJ.

[PDF_00523] Widell A., Hansson B. G., Berntorp E., Moestrup T., Johansson H. P., Hansson H., Nordenfelt E. (1991). Antibody to a hepatitis C virus related protein among patients at high risk for hepatitis B.. Scand J Infect Dis.

[PDF_00543] van den Hoek J. A., van Haastrecht H. J., Goudsmit J., de Wolf F., Coutinho R. A. (1990). Prevalence, incidence, and risk factors of hepatitis C virus infection among drug users in Amsterdam.. J Infect Dis.

